# Three-dimensional analysis of the velopharyngeal region in patients with cleft palate and healthy individuals

**DOI:** 10.1007/s00276-020-02526-3

**Published:** 2020-07-08

**Authors:** Simone Miller, Michael-Tobias Neuhaus, Rüdiger Zimmerer, Frank Tavassol, Nils-Claudius Gellrich, Martin Ptok, Michael Jungheim

**Affiliations:** 1 Department of Phoniatrics and Pediatric Audiology, Hannover Medical School, Carl-Neuberg-Street 1, 30625 Hannover, Germany; 2 Department of Oral and Maxillofacial Surgery, Hannover Medical School, Hannover, Germany

**Keywords:** Cleft palate, Velopharyngeal dimensions, Cone beam computed tomography, Velopharynx

## Abstract

**Purpose:**

This study aims to attain metric data of the velopharyngeal dimensions of healthy subjects as well as patients with velopharyngeal insufficiency using the example of cleft and lip palate (CLP) in order to determine possible differences in the volumes of both groups.

**Methods:**

Volumes and distances of velopharyngeal areas were analyzed retrospectively using cone beam computed tomography data sets (*n* = 60). Group 1 included healthy patients receiving dental implants (*n* = 31). Group 2 was represented by patients with surgically closed cleft lip and palate (*n* = 29).

**Results:**

Biggest differences among mean values of both groups were found for: minimum axial area (*p* = 0.000), airway area caudal (*p* = 0.000), distance between posterior nasal spine and posterior pharyngeal wall (PPW) (*p* = 0.014), mean distance between velum and PPW (*p* = 0.000), length of PPW (*p* = 0.000) and length of anterior pharyngeal wall (*p* = 0.000).

**Conclusion:**

Differences in the shape and geometry of the velopharyngeal area in subjects with a regular velopharyngeal structure and function and patients with cleft palate do exist. The significant differences found here can be categorized into two groups: one reflects distances between the anterior and posterior pharynx, presenting longer distances for patients with CLP. The second significant difference regards values of length in cranio-caudal direction, which is longer in healthy subjects. With regards to these values, one could conclude, that even though total volumes of both groups did not differ in size, group 1 shows three-dimensional velopharyngeal shapes that are longer and narrower, whereas shapes of patients of group 2 tend to be wider and shorter in general.

## Introduction

The velopharyngeal closure represents a very important function during different tasks, such as swallowing and speech. In both swallowing as well as phonation the muscles of the pharynx and the soft palate are involved fundamentally [8], by opening and closing the airway between the naso- and oropharynx (velopharyngeal closure). The velopharyngeal closure is achieved by the velum, lateral and posterior pharyngeal walls. Depending on the exact task the following muscles may be involved: m. levator veli palatini, m. tensor veli palatini, m. constrictor pharyngis superior, m. palatopharyngeus and m. palatoglossus [8, 9]. During swallowing the velopharyngeal closure prevents food regurgitation into the nasal cavity, and during phonation it regulates nasal resonance. Velopharyngeal insufficiency (VPI) may result in dysphagia as well as speech impairments, such as rhinophonia [8].

A variety of diseases, for example neurogenic movement disorders such as amyotrophic lateral sclerosis or myasthenia gravis, congenital diseases such as cleft palate or postoperative conditions, for example after tonsillectomy, can lead to velopharyngeal insufficiency [8].

The spatio-temporal pattern of muscle actions and the resulting volume, as in the shape of the cavities in the naso- and velopharynx resulting from movements of the structures involved during various tasks has not been fully determined yet.

Due to its excellent tissue contrasting against air, cone beam computed tomography (CBCT) allows for reliable high resolution 3D airway segmentation [6]. CBCT has become a standard diagnostic tool in dentistry and oral and maxillofacial surgery and has especially gained importance for computer assisted planning and design as well as implant dentistry [4, 7, 12]. With an equal sub-millimeter resolution, it uses up to 15 times less radiation dose than a multi slice CT [14] and delivers comparable information on different anatomical structures [13]. CBCT is generating large numbers of datasets, allowing for 3D assessment of velopharyngeal configuration.

In a first step this study aims to attain metric data of the velopharyngeal dimensions of healthy subjects as well as patients with velopharyngeal insufficiency (here surgically closed cleft palate) at rest using CBCT in order to determine whether differences in dimensions exist with regard to clinical significance. In addition, the data might be useful to provide a basis for the development of therapeutical devices as well as therapeutical strategies for the treatment of VPI, which require a metric analysis.

## Materials and methods

### Study design

Single center, retrospective cohort study.

### Ethical approval

The retrospective analysis of patient data was approved by the institution’s ethics committee (22.02.2019). All patient data had been collected as part of clinical routine assessments and not for study related activities.

### Patients

CBCT data sets of 60 patients of the Department of Oral and Maxillofacial Surgery were analyzed. Patients were part of two patient groups. Group 1 included healthy patients receiving dental implants (*n* = 31, 14 males 17 females, ages 36–80), without any further velopharyngeal functions being affected. The other patient group was represented by patients with cleft lip and palate (*n* = 29, 15 males, 14 females, ages 6–61), who underwent cleft surgery in their early childhood. For both groups CBCT of the head and neck was performed routinely before dental implantation. Data sets were selected from a reference date on (February 2019), going backwards in time, 30 data sets were aimed for.

### Cone beam computed tomography

In this study CBCT scans were taken using a Vatech PaX-Zenith 3D^®^ (13, Samsung 1-ro 2-gil, Hwaseong-si, Gyeonggi-do, 445-170, Korea). The device has a voxel size of 0.3 mm. Patient scans have been performed with a small occlusal gap and they were told not to move their heads or tongues during scanning. Dose area product of standard CBCT scan was 8.96 dGy × cm^2^.

### Dolphin 3D analysis software

**Fig. 1:**
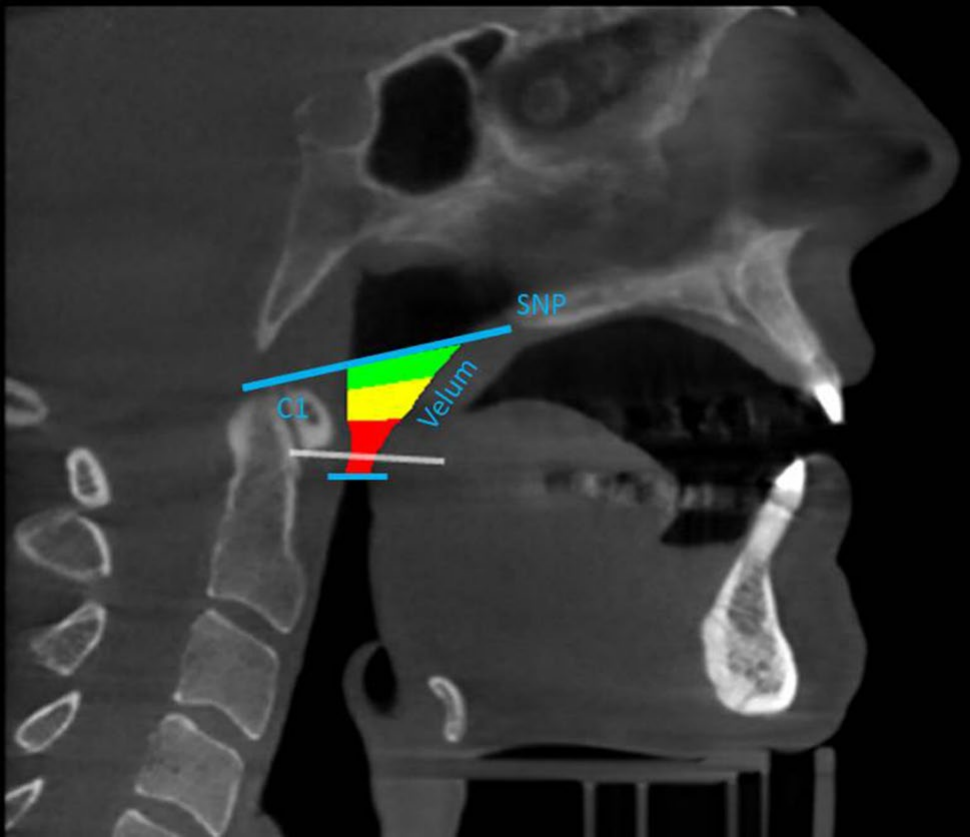
Definition of the three-dimensional velopharyngeal area: upper border defined by a straight line between the posterior nasal spine (PNS) and the upper limit of the atlas (C1). The lower border is set to the uvula/end of the soft palate

Dolphin 3D is a virtual planning software for orthognatic surgery. With a 3D patient dataset, e.g., CT or CBCT, a surgical treatment plan can be generated. Furthermore this software allows for segmentation, visualization and measurement of upper airway spaces like naso-, oro- and laryngopharynx as well as the nasal cavity (Dolphin Imaging and Management Solutions 9200 Oakdale Ave. Suite 500 Chatsworth, CA, 91311, USA). After determination of the margins of this airway segmentation (see Fig. 1), its volume and dimensions are automatically delivered by the software. More specific dimensions between anatomical landmarks can be measured manually. Analysis of all patient data sets has been performed by the same investigator.

### Parameter definition

Patient datasets have been aligned according to the Frankfurt horizontal plane. The airway is segmented by the following margins (see Fig. 1) in the mid-sagittal plane view of the CBCT slice. The upper border is defined by a straight line reaching from the posterior nasal spine (PNS) and the posterior pharyngeal wall (PPW) at the height of the upper limit of the atlas (C1). The lower border of the airway is defined as the end of the soft palate to the posterior pharyngeal wall at the same height. During this study the end of the velum was chosen in mid-sagittal view. The most caudal point on the velum was chosen which did not result in expanding the volume into the oral cavity.

**Fig. 2:**
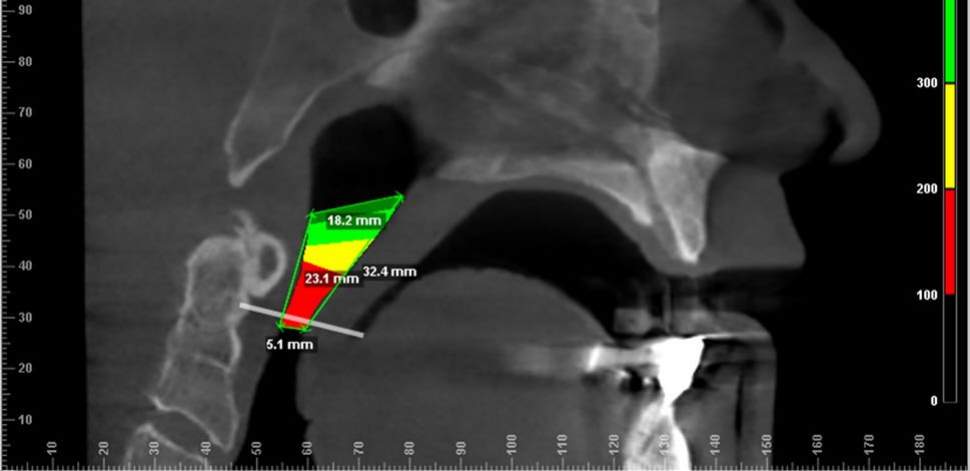
Margins of velopharyngeal airway segmentation in sagittal plane view. Distance PNS-PPW measures the distance between the posterior nasal spine and the pharyngeal wall at the height of the upper limit of the atlas (C1) (here 18.2 mm). Distance velum-PPW measures the distance between the lower border of the end of the soft palate to the posterior pharyngeal wall at the same height (here: 5.1 mm). Length of posterior pharyngeal wall measures the distance between the pharyngeal wall at the height of the upper limit of the atlas (C1) and the pharyngeal wall at the height of the lower border of end of the soft palate (here: 23.1 mm). Length of anterior pharyngeal wall measures the distance between PNS and lower border of end of the soft palate (here: 32.4 mm)

In the mid-sagittal plane view (see Fig. 2).

**Fig. 3:**
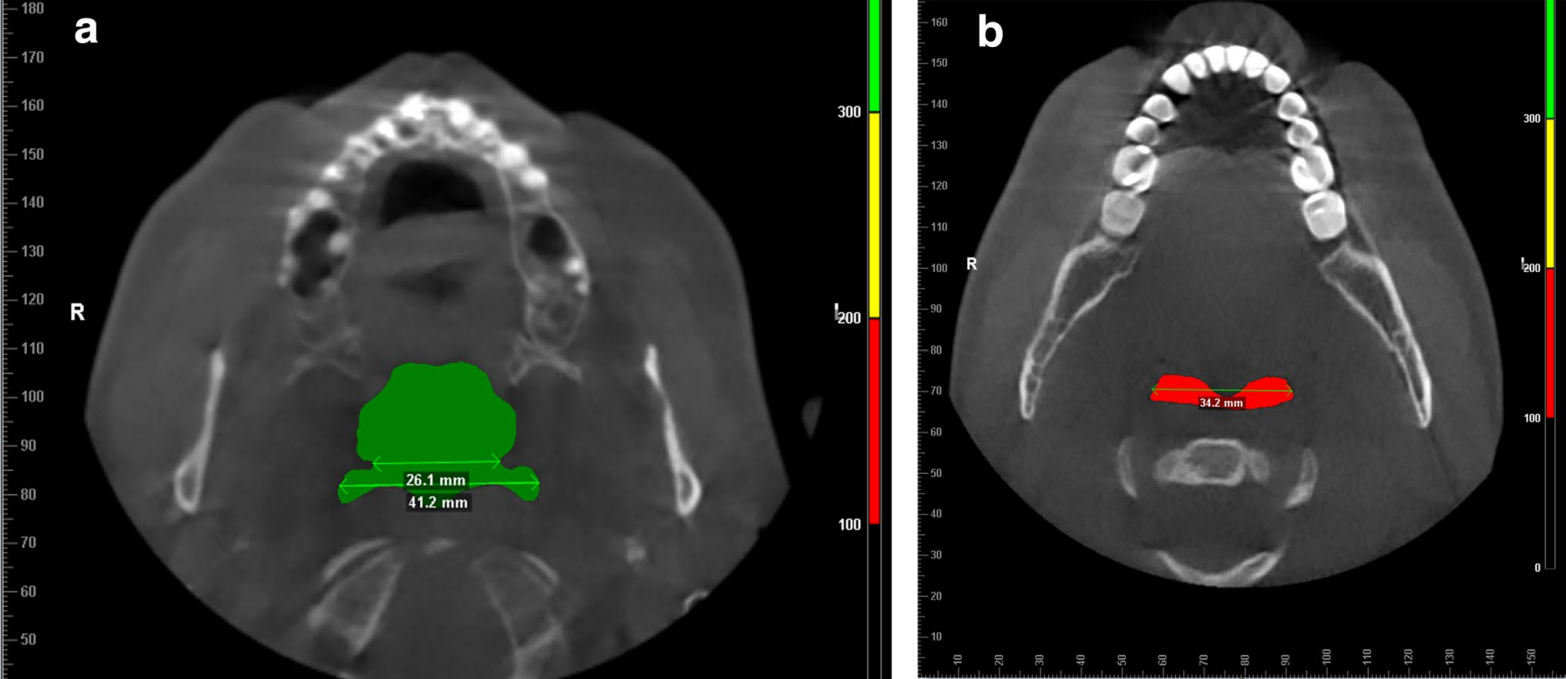
**a** Parameters attained in transverse plane view: cranial area slice of selected airway volume, distance between auditory tubes (here 26.1 mm), maximal distance (here: 41.2 mm). **b** Parameters attained in transverse plane view: caudal area slice of selected airway volume and maximal distance (here: 34.2 mm).

- Airway area (mm^2^)
  Measures the area of the selected volume (see above).
- Distance PNS-PPW
  Measures the distance between the posterior nasal spine (PNS) and the posterior pharyngeal wall (PPW) at the height of the upper limit of the atlas (C1).
  - Cranial border for calculation of velopharyngeal volume.
- Distance velum-PPW
  Measures the distance between the end of the soft palate to the posterior pharyngeal wall at the same height.
  - Inferior border for calculation of velopharyngeal volume.
- Length of posterior pharyngeal wall
  Measures the distance between the pharyngeal wall at the height of the upper limit of the atlas (C1) and the pharyngeal wall at the height of the lower border of the uvula/end of the soft palate.
- Length of anterior pharyngeal wall
  Measures the distance between PNS and lower border of the uvula/end of the soft palate.
  In transverse plane view:
- Minimum axial area (mm^2^)
  Measures the minimal area within the selected volume.
- Airway volume (mm^3^)
  Measures the entire selected volume.
- Airway area cranial (mm^2^)
  Most cranial slice of segmented airway volume (see Fig. 3a).
- Cranial area, distance between auditory tubes (mm) (see Fig. 3a)
- Cranial area, maximal distance (mm) (see Fig. 3a)
- Airway area caudal (mm^2^)
  Most caudal slice of segmented airway volume (see Fig. 3b).
- Caudal area maximal distance (mm) (see Fig. 3b)

### Statistical analysis

Data was analyzed using IBM Statistics SPSS (Version 25.0; IBM Corp, Armonk, NY, USA). A *t *test for independent samples was carried out comparing parameters between both patient groups. Additionally, these comparisons were confirmed by excluding patients under the age of 18 (minors of group 2 and adults of group 2). Furthermore gender differences, differences between UCLP and BCLP, as well as differences in relation to rapid maxillary extension (RME) were determined.

## Results

Patients (*n* = 60) showed an average age of 40 years (median 41 years). Group 1 (*n* = 31) presented with an average age of 62 (median 63 years), and group 2 (*n* = 29) an average age of 18 years (median: 17 years). Excluding all patients below the age of 18, patients (*n* = 44) presented with an average age of 51 years (median 58 years), group 1 (*n *= 31) averaged at 62 years (median 63 years), group 2 (*n* = 13) averaged at 24 years (median 21 years). Mean values for all parameters are shown in Table 1 and *p *values from statistical comparisons are shown in Table 2.

**Table 1:** Mean values of all parameters across different patient groups

	*n*	Age		Airway area (mm^2^)		Minimum axial area (mm^2^)		Airway volume (mm^3^)		Airway area cranial (mm^2^)		Airway area caudal (mm^2^)		Saggital distance PNS-PPW (mm)		Saggital, distance velum-PPW (mm)		Saggital, length of posterior pharyngeal wall (mm)		Saggital, length of anterior pharyngeal wall/velum		Transverse, cranial area distance between auditory tubes		Transverse, cranial area maximal distance		Transverse, caudal area maximal distance	
		Mean	Median	Mean	SD ±	Mean	SD ±	Mean	SD ±	Mean	SD ±	Mean	SD ±	Mean	SD ±	Mean	SD ±	Mean	SD ±	Mean	SD ±	Mean	SD ±	Mean	SD ±	Mean	SD ±
Implant																											
All	31	62	63	201.29	65.07	155.94	76.51	5877.0	2367.86	443.87	135.95	176.13	59.08	19.36	3.66	5.35	2.1	20.69	4.74	27.65	3.86	26.95	4.47	38.10	8.27	28.93	5.98
Male	14	63	66	223.21	56.38	163.00	57.95	6964.79	1806.47	496.71	107.05	181.50	45.24	20.27	3.26	5.09	1.84	22.23	3.85	29.72	4.26	28.69	3.49	41.24	6.93	30.57	5.19
Female	17	60	60	183.24	67.74	150.12	90.37	4981.18	2444.01	400.35	144.53	171.71	69.54	18.61	3.90	5.57	2.33	19.42	5.13	25.94	2.52	25.52	4.78	35.18	8.67	27.58	6.40
CLP																											
All	29	18	17	210.72	58.97	302.27	133.07	5609.90	2065.27	475.48	145.23	312.69	139.21	21.88	4.01	11.61	3.41	14.02	3.42	21.28	4.29	26.41	6.10	35.22	7.70	30.36	8.56
Male	15	19	14	219.80	63.11	288.13	111.61	5485.47	2201.89	427.40	136.03	299.27	119.22	21.33	3.57	12.91	2.77	14.26	3.06	20.57	3.39	25.87	6.00	34.03	8.53	28.99	7.64
Female	14	17	18	201.00	54.82	317.00	160.06	5743.21	1982.09	527.00	141.33	327.07	161.26	22.47	4.50	10.21	3.56	13.76	3.86	22.04	5.11	26.99	6.38	36.49	6.78	31.83	9.51
Adults	13	24	21	204.08	66.79	336.23	128.04	5886.15	2233.95	509.38	146.05	350.23	125.15	22.04	4.13	11.36	3.33	14.06	3.79	21.69	3.59	28.12	6.63	37.79	6.68	33.73	8.79
ULCLP	17			214.65	61.50	344.18	130.77	5978.06	1973.11	505.00	142.17	367.00	129.74	22.14	3.80	12.28	3.47	14.15	3.63	21.15	3.69	28.15	6.19	36.79	7.54	33.58	8.24
BLCLP	8			209.50	65.38	271.38	138.81	5432.25	2444.11	495.00	135.91	264.13	127.65	23.46	4.05	10.64	2.66	12.81	3.31	21.08	6.20	26.10	4.45	33.99	8.02	27.26	7.40
RME Yes	3			222.3	62.58	259.33	177.06	5832.67	2792.22	543.33	191.75	253.00	150.14	25.27	5.42	9.83	3.09	9.83	3.09	23.90	8.70	25.90	2.31	36.47	7.24	28.00	8.25
RME No	26			209.38	59.70	307.00	133.36	5584.19	2036.38	467.65	141.65	319.58	139.38	21.49	3.76	11.82	3.44	14.10	3.44	20.98	3.70	26.47	6.42	35.07	7.88	30.64	8.71

*CLP* cleft lip palate, *UCLP* unilateral cleft lip palate, *BCLP* bilateral cleft lip palate, *RME* rapid maxillary expansion, *PNS* posterior nasal spine, *PPW* posterior pharyngeal wall)

**Table 2:** *p* values for pairwise statistical comparisons

	Airway area (mm^2^)	Minimum axial area (mm^2^)	Airway volume (mm^3^)	Airway Area cranial (mm^2^)	Airway Area caudal (mm^2^)	Saggital distance PNS-PPW (mm)	Saggital, distance velum-PPW (mm)	Saggital, length of posterior pharyngeal wall (mm)	Saggital, length of pharyngeal wall/velum anterior	Transverse, cranial area distance between auditory tubes	Transverse, cranial area maximal distance	Transverse, caudal area maximal distance
	*p *value	*p *value	*p *value	*p *value	*p *value	*p *value	*p *value	*p *value	*p *value	*p *value	*p *value	*p *value
Statistical comparison (*p *value)												
Group 1 vs. group 2	0.559	0.000**	0.644	0.387	0.000**	0.014*	0.000**	0.000**	0.000**	0.697	0.175	0.460
Group 1 vs. group 2 adults only	0.898	0.000**	0.991	0.161	0.000**	0.039*	0.000**	0.000**	0.000**	0.501	0.904	0.041*
Adults vs. minors of group 2	0.314	0.804	0.739	0.817	0.787	0.587	0.671	0.786	0.124	0.383	0.383	0.748
Group 1: male vs. female	0.089	0.649	0.018	0.048	0.654	0.213	0.536	0.102	0.008	0.048*	0.047	0.170
Group 2: male vs. female	0.401	0.576	0.744	0.064	0.600	0.460	0.031	0.700	0.369	0.630	0.401	0.382
Group 2 ULCLP vs. BLCLP	0.850	0.215	0.555	0.869	0.076	0.435	0.249	0.386	0.974	0.412	0.404	0.078
Group 2 RME vs. no RME	0.726	0.573	0.848	0.403	0.443	0.125	0.350	0.719	0.272	0.882	0.773	0.623

*CLP* cleft lip palate, *UCLP* unilateral cleft lip palate, *BCLP* bilateral cleft lip palate, *RME* rapid maxillary expansion, *PNS* posterior nasal spine, *PPW* posterior pharyngeal wall

*Marks values of statistical significance and **values of high statistical significance

### Airway area (mm^2^)

Group 1 presented with a slightly smaller mean airway area than group 2. These differences did not show statistical significance (*p* = 0.559).

### Minimum axial area (mm^2^)

Group 1 showed an average minimum axial area of 155.94 mm^2^ (±76.51) and group 2 of 302.27 mm^2^ (±133.07 mm^2^). These differences revealed high statistical significance between both groups (*p* = 0.000).

### Airway volume (mm^3^)

Group 1 presented with a slightly larger average airway volume than group 2. Differences in relation with airway volume did not show statistical significance (*p* = 0.644).

### Airway area cranial (mm^2^)

The mean cranial airway area differences did not show statistical significance in any of the comparisons (see Table 2).

### Airway area caudal (mm^2^)

Average caudal airway areas also were larger in group 2 (312.69 mm^2^ ± 139.21 mm^2^) than group 1 (176.13 mm^2^ ± 59.08 mm^2^). These differences were highly statistically significant (*p* = 0.000).

### Sagittal distance PNS-PPW

Average distances between the posterior nasal spine and the posterior pharyngeal wall measured longer in group 2 (21.88 mm ± 4.01 mm) than in group 1 (19.36 mm ± 3.66 mm). These differences revealed statistical significance (*p* = 0.014).

### Sagittal, distance velum-PPW

Also, the mean distance between the velum and the posterior pharyngeal wall (PPW) measured longer in group 2 (11.61 mm ± 3.41 mm) than group 1 (5.35 ± 2.1 mm). These differences showed high statistical significance (*p* = 0.000).

### Sagittal, length of posterior pharyngeal wall

The mean length of the measured area of the posterior pharyngeal wall presented to be longer in group 1 than group 2. Subjects of group 1 measured average distances of 20.69 mm ± 4.74 mm and patients in group 2 measured 14.02 mm ± 3.42 mm. These differences also showed high statistical significance (*p *= 0.000).

### Sagittal, length of anterior pharyngeal wall

The same area of the anterior pharyngeal wall measured 27.65 mm ± 3.86 mm in group 1, and 21.28 mm ± 4.29 mm in group 2 on average. This difference also reached high statistical significance (*p* = 0.000).

### Transverse, cranial area distance between auditory tubes

Mean distances in the transverse plane between both auditory tubes measured almost identical for both groups. These differences did not show statistical significance (see Table 2).

### Transverse, cranial area maximal distance

Differences among mean maximum distances in the transverse plane of the cranial area did not show statistical significance (see Table 2).

### Transverse, caudal area maximal distance

Differences between mean maximum distances in the transverse plane of the caudal area did not show statistical significance.

### Age group comparisons

Due to an inhomogeneity of the two groups in age two further comparisons were implemented. Firstly, all minors and adults of group 2 were statistically compared. No statistically significant differences were found. Secondly, statistical comparison was repeated considering the data of adults only (group 1 vs. adults of group 2). Comparisons indicated the same statistically significant differences regarding the parameters as the two entire group comparisons did (see Table 2).

### Gender differences

Comparisons on male vs. female subjects within both groups (group 1, group 2) revealed differences for certain parameters. For the group of implanted patients, statistically significant differences were found for airway volume (*p* = 0.018), airway area (*p* = 0.048), sagittal length of anterior pharyngeal wall/velum (*p* = 0.008), transverse, cranial area distance between auditory tubes (*p* = 0.048) and transverse, cranial area maximal distance (*p* = 0.047), whereas in the cleft and lip palate (CLP) group the only difference was to be found for the distance between the velum and the posterior pharyngeal wall (*p* = 0.031).

### Comparison between unilateral (ULCLP) and bilateral cleft (BLCLP)

Comparisons between individuals of group 2 (CLP) with ULCLP (*n* = 17) vs. BLCLP (*n* = 8) revealed no statistical differences for any of the tested parameters (see Table 2).

### Comparison between RME and no RME

Group 2 included three patients with a rapid maxillary expansion (RME) prior to the CBCT-scan. No statistical differences were seen between patients with or without RME (see Table 2).

### Inter-rater and intra-rater agreement

The inter-rater agreement as calculated with the intra class correlation coefficient measured from 0.835 to 0.998 for all parameters. According to Koo and Li [11] this is indicative of good to excellent reliability. The intra class correlation coefficient for the intra rater agreement measured from 0.746 to 1.000 across the parameters, which is indicative of a moderate/good to excellent reliability according to Koo and Li [11], and good/excellent to excellent according to Cicchetti [5].

## Discussion

**Fig. 4:**
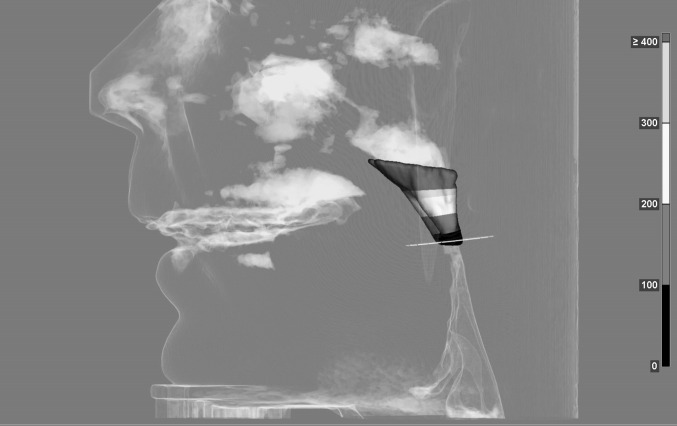
Three-dimensional volume of the velopharynx of a healthy subject

**Fig. 5:**
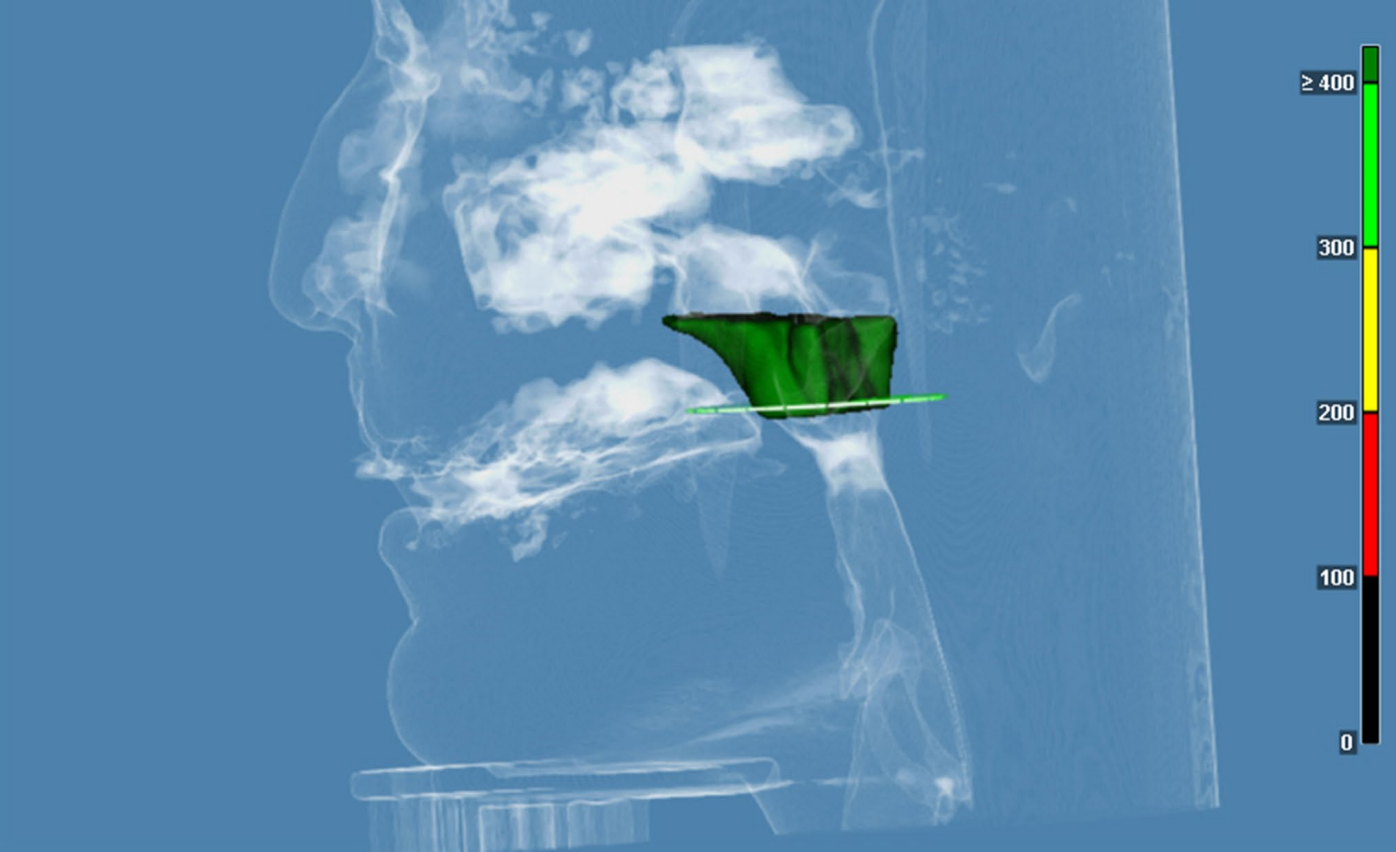
Three dimensionasl volume of the velopharynx of a patient with bilateral cleft lip and palate

This study aimed to attain metric data of the velopharyngeal dimensions of healthy subjects as well as patients with velopharyngeal insufficiency and determine whether differences in dimensions exist with regard to clinical significance. It becomes evident, that there are differences in the shape and geometry of the velopharynx in subjects with a regular velopharyngeal structure and function and patients with cleft lip and palate. The significant differences found here can be categorized into two findings: one regards the measurement of the velopharynx between the anterior and posterior pharynx showing longer distances for patients with cleft lip and palate. The second significant difference regards values of length in a cranio-caudal direction. These are longer in healthy subjects, confirming that the soft palate is longer in healthy subjects than in patients with CLP. With regards to these values, it can be concluded that group 1 shows three-dimensional velopharyngeal shapes that are longer and narrower, whereas shapes of patients of group 2 tend to be wider and shorter in general (see Figs. 4, 5).

Due to the treatment plan regarding surgery as well as dental implantation CBCT in patients with cleft lip and palate is performed at relatively young age. The age span of group 2 ranged from 6 to 61 (whereas there was only one patient at age 61 the next younger patient was 25). Since some differences in sizes and dimensions of the velopharynx might be reflected by a difference in age, two measures were implemented during this study. First of all minors and adults of group 2 were statistically compared for differences. Since no statistically significant differences were found and since, 85–95% of the human skull growth occurs before the age of ten [15] a comparison of all subject and patient data was justified. Nevertheless statistical comparison was repeated considering the data of adults only. Both comparisons indicated the same statistically significant differences regarding the parameters. This enabled a comparison between the two main groups 1 and 2.

A previous study by Celikoglu et al. found oropharyngeal airway volume to be lower in ULCP patients than in a control group [1]. No difference in total airway volume was obtained, similar to our study. Another study comparing the airway volume of patients with bilateral cleft lip and palate (BLCP) and a healthy control group found total airway volumes to be lower in BLCP patients compared to healthy controls, as well [2]. No differences in total airway volumes, as well as cross sectional areas between patients with CLP and controls were reported by Cheung et al. [3]. As study protocols and airway sectioning differed between this study and those studies mentioned, a comparison between data is possible only to a limited degree. However, placement of the inferior border of airway segmentation at the end of the soft palate might be more expedient in order to obtain cleft related differences, since in the oro- and laryngopharyngeal region down to the epiglottis, the anatomy should not be affected by the existence of a cleft. As former studies may be biased due to patient selection, sample size and varying therapy plan [1], in this study the same measures as described above for patient’s age have been performed for ULCP and BLCP patients. No significant differences between ULCP and BLCP with regards to airway volume and dimension have been found. Three patients with an RME prior to CBCT-scan have been included in this study. However, a bias could be ruled out, since no statistical correlation between RME and airway volume and dimension was seen (see Table 2).

We, therefore, conclude that a difference in anatomic structures persists in patients with cleft palate even after surgical closure. It can be assumed that the dimensional differences shown here also have an influence on the velopharyngeal function [10]. This, however, is subject to further studies.

## Limitations

Limitations of this study are given by the inhomogeneity of the groups with regards to age, cleft-type or treatment. Furthermore the retrospective study design did not allow for sufficient assessment of velopharyngeal proficiency prior to patient selection. This study design did also not allow to determine degrees of velopharyngeal function or insufficiency. This should be considered in future studies.

## Conclusions and outlook

Differences in shape and dimension between CLP patients and healthy individuals could be shown. CLP patients present with a wider but shorter velopharyngeal region than healthy individuals. No differences exist between ULCP and BLCP patients for this matter. This might be helpful to put the contrary findings in literature in order. Further work should focus on establishing a shape model of the velopharynx to analyze velopharyngeal functions with regard to rhinophonia as well as optimize possible stimulation approaches and applicators for this region.
